# The Effect of Connected “Smart” Inhalers on Medication Adherence

**DOI:** 10.3389/fmedt.2021.657321

**Published:** 2021-08-18

**Authors:** Caroline Zabczyk, John D. Blakey

**Affiliations:** ^1^Respiratory Medicine, Sir Charles Gairdner Hospital, Perth, WA, Australia; ^2^Medical School, Curtin University, Perth, WA, Australia

**Keywords:** asthma, chronic obstructive pulmonary disease, inhaled administration, adherence, digital technology, smart inhalers

## Abstract

Asthma and chronic obstructive pulmonary disease (COPD) are highly prevalent worldwide, and major sources of morbidity. Key barriers to reduce the harm from these conditions are the widespread and related issues of low use of prescribed inhaled therapy, use of medicines differently from that prescribed, suboptimal inhaler technique, and lack of adherence are the action plans. Connected smart inhalers show great potential to improve these issues, and thus outcomes from airways disease. In this mini-review, we considered the published evidence that the use of smart inhalers leads to more doses of preventative treatment being taken on time and with appropriate techniques. We found multiple trials across a variety of settings and age groups where smart inhalers were used with audio-visual reminders and healthcare professional feedback, which substantially improved the number of doses of preventative treatment taken. Trial evidence also supports the use of feedback from smart inhalers in improving true concordance (doses taken correctly and on time), though only for a single type of smart device. The relative lack of study is in contrast with the potential impact of smart inhalers. Major research questions remain unresolved, as who might fund future large-scale studies, how guideline committees may consider them, and how to implement effective solutions.

## Introduction

Obstructive airways diseases affect people of all ages worldwide. These conditions are highly prevalent, for example in Australia, more than 10% of the population have asthma, and ~5% aged 45 and over have chronic obstructive airways disease (COPD) (1, 2). On a larger scale, asthma was estimated to affect 262 million people worldwide and caused 461,000 deaths in 2019 (3). COPD is the third leading cause of death worldwide, with 3.23 million deaths in 2019 (4). Best practice treatment guidelines for asthma and COPD are well-established and accepted [e.g., (5)]. Despite the multitude of clinically effective treatment options available, there is persistence in poor outcomes for patients with asthma and COPD. Unscheduled healthcare visits and impaired quality of life remain common (6, 7). These issues lead to tremendous direct healthcare costs and indirect costs of a similar magnitude (8, 9). According to the AIHW Disease Expenditure Database, in 2015–2016, asthma cost the Australian Health system an estimated $770 million, and COPD cost US$977 million (1, 2).

### Adherence

As with other chronic diseases, suboptimal adherence to therapy is a key driver of the observed high morbidity, mortality, healthcare expenditure, and decreased quality of life in patients with

asthma and COPD (10, 11). Adherence is defined by the WHO as “the degree to which the person's behaviour corresponds with the agreed recommendations from a health care provider.”

**Figure 1:**
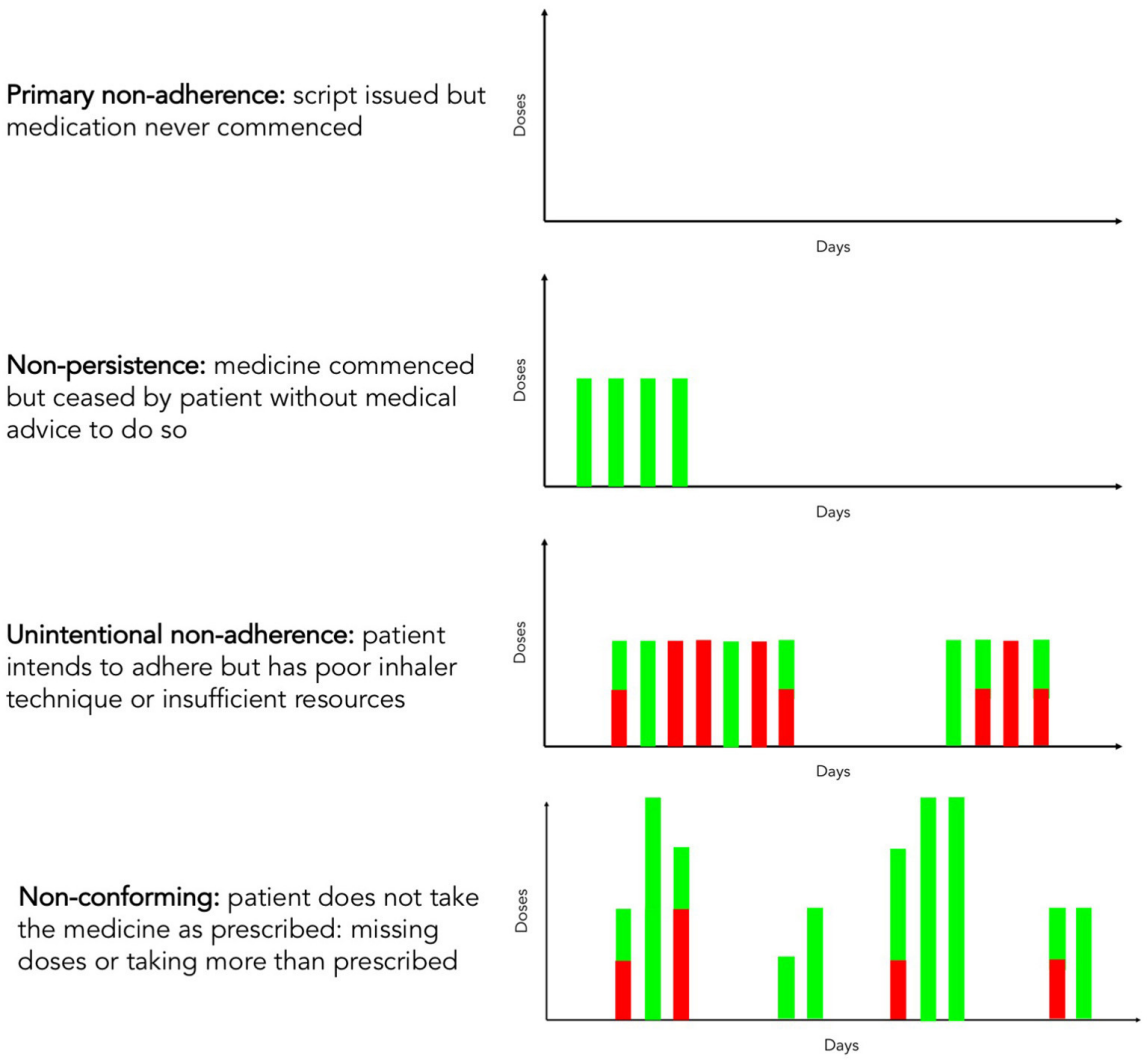
Adherence and competence with respect to inhaler use. Green columns represent inhaler doses correctly administered by the patient, and red columns represent missed or incorrectly administered doses due to poor inhaler technique, insufficient resources or non-adherence.

Non-adherence can be variously classified for clinical and research purposes (12). The main categories of adherence issues are shown in Figure 1. It has long been clear that a step change is required in all these adherence categories in relation to inhaled medication for airways disease. A minority of individuals possess sufficient medication to take their inhalers as prescribed, with a variety of drivers for this (13). Those that collect their inhalers often have suboptimal inhaler techniques, with common types of errors being associated with adverse clinical outcomes. The proportion of appropriately taken doses in the overall number of inhalations may be referred to as *competence* (14). Additionally, inhalers are commonly discarded before they are empty (15). No intervention has been shown to improve all aspects of adherence to inhaled therapy at scale.

Given the burden and cost of airways diseases and the relative lack of progress with inhaler concordance, the emergence of “smart” connected inhalers is a potentially crucial step forward in this space.

### Smart Inhalers and Their Potential

**Figure 2:**
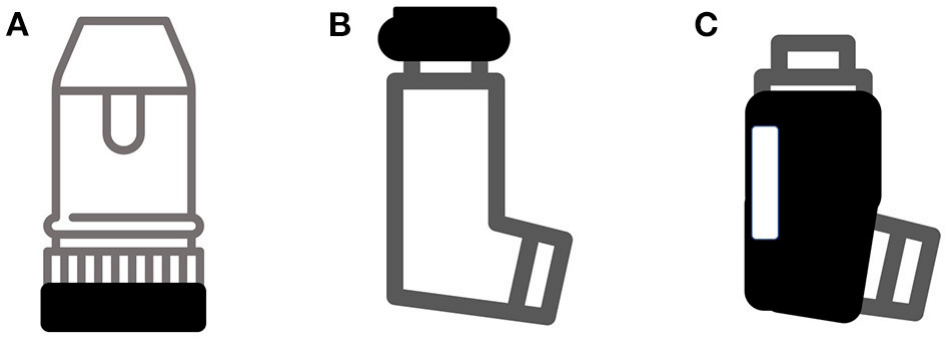
Common types of add-on devices to create smart inhalers. **(A)** Detection of device priming; **(B)** Detection of patient actuating device; **(C)** Jacket to detect airflow. Not shown are devices such as INhaler Compliance Assessment (INCA) that detect actuation and technique purely acoustically.

The term “smart inhaler” is not subject to a universally agreed definition. As a working description, we consider them to be inhaler-based electronic monitoring systems to record drug usage, with or without an assessment of the technique of device use. This practical characterisation does not include nebulisers, or software systems that purely use a separate electronic device e.g., image recognition technology on phones or tablets. The majority of smart inhalers deployed over the past decade have been of the format of a standard inhaler with an additional electronic fitment. Examples of this are demonstrated in Figure 2.

Electronic monitoring of inhaler adherence has been available for more than 30 years, with the *Nebuliser Chronolog* (16) being the first device in common academic use. However, it is only in the past 10 years or so that the relevant electronics have become sufficiently small, reliable, and affordable to realise the potential for widespread deployment. These advances have been accompanied by improvement in wireless technologies and mobile computing that allow information to be rapidly transferred from the inhaler, remotely analysed, on a personal device, and presented to the user in a meaningful and graphically pleasing manner.

The technological capabilities available currently include recording when an inhaler was used, reminding patients of their next dose, analysis of technique through measurement of inspiratory flow rate, and feedback on technique and timing of inhaler use. The data generated by these devices can also be transferred securely to a healthcare professional to provide a “real world” perspective on adherence and competence to medication of each patient (17). This adds further layers of potential benefit in terms of adherence: the user knows their activities are being recorded and so may be more concordant, and the healthcare practitioner may tailor their consultations to the individual patient with these data to promote regular appropriate use or recognise and praise concordance. Furthermore, the concordance amongst each doctor or team's patients is also known, allowing peer benchmarking.

### Barriers to the Implementation of Smart Inhalers

There is resistance to change the established patterns of working in all areas of medicine even when well-established guidance is available (18). There are additional issues in relation to smart inhalers are around:

- the acceptability of this monitoring to patients who may feel their autonomy and privacy is compromised
- uncertainty around clinical responsibility for reviewing and acting on these new large-scale continuous data streams
- lack of clarity over who will bear the cost of new technologies.

The majority of perceived and actual barriers can be overcome by high-quality research publications demonstrating the efficacy and effectiveness of “smart inhalers” in improving adherence, disease monitoring, and ultimately health outcomes for patients with asthma and COPD. Once effectiveness is established, a clear framework can be constructed to integrate their use into clinical practise guidelines. This review, therefore, aims to summarise the available evidence that the use of smart inhalers increases true medication adherence.

## Review Framework

This mini-review considers the following potential opportunities for connected smart inhalers to improve adherence:

- Increase the number of doses of preventer taken (total doses)
- Increase the number of doses of preventer taken as prescribed (doses taken on time)
- Increase the number of doses of preventer taken appropriately (doses taken with good technique)
- Facilitation of appropriate escalation to next step in asthma or COPD action plan (minimise harm if it is occurring).

The review is of a narrative type rather than a systematic evaluation given the paucity of trial evidence and the heterogeneity in studies that have been undertaken.

## Smart Inhalers and Concordance With Regular Inhaled Therapy

### Do Smart Inhalers Increase Medicine Use?

Studies in this area have considered the value of audio-visual reminder feedback (AVRF) from smart inhalers, and the provision of healthcare professional feedback in the clinic, to promote adherence.

In 2007, Charles et al. (19) published the results of a study that had enrolled 110 individuals with asthma aged 12–65 into a randomised trial of AVRF. All participants received a smart inhaler, but in the control arm, it was solely used to capture the primary outcome of medicine adherence. There was a significantly greater use of medicine in the intervention group than the control group during the second of 12 weeks of follow up (88% of total intended doses vs. 66%). The overall high–rate of concordance may reflect participants being recruited from a database of research volunteers. A subsequent larger randomised trial replicated these findings in a different setting (20). Similar to the Charles study, Chan et al. provided smart inhalers to participants but only the intervention arm had AVRF activated. Importantly, this study recruited only children and adolescents from those attending acutely with a suspected exacerbation. Over the 6-month follow-up, adherence was significantly higher in the group receiving reminders (median 84% of total intended doses) than those who did not (30%).

The effect of healthcare professionals feeding back concordance information from smart inhalers was investigated by Burgess et al. (21) in individuals attending a paediatric respiratory clinic. In this randomised pilot study (total *n* = 26), medication adherence was higher in those children receiving feedback (79%) than those that did not (58%) over 4 months.

Combining the potential of AVRF and feedback from healthcare professionals, the “STudy of Asthma Adherence Reminders” (STAAR) (22) enrolled children with uncontrolled asthma (Asthma Control Questionnaire >1.5). All children received a smart inhaler, but the intervention group was told their data would be downloaded and reviewed at the clinic, and their devices also provided audible reminders of doses due. There was a substantial difference in adherence, albeit this was a secondary outcome: 70% of total intended doses were dispensed in the intervention group vs. 49% in the control group. These results should be interpreted in the context of 50% of the intervention group reporting their devices to be broken, compared to 19% in the control arm. This phenomenon has been alluded to but not so clearly demonstrated in other studies.

Complementing and extending these secondary care paediatric results, Foster et al. (23) undertook a cluster randomised trial amongst adolescents and adults in primary care. Sixty general practitioners (GPs) were randomly selected to provide either inhaler reminders and feedback, personalised adherence discussions, both of these interventions, or usual care. The 40 GPs completed the study enrolling 143 patients on LABA-ICS with suboptimal asthma control (Asthma Control Test <19). Those individuals in the AVRT groups took around twice as great a proportion of the prescribed doses of preventer at 6 months of follow-up (60 vs. 29%).

Therefore, as demonstrated in the studies carried out thus far, smart inhalers do have the potential to increase adherence to inhaled medication use through AVRF and by providing feedback to healthcare professionals. However, further large-scale studies specific to testing smart inhaler efficacy are required to provide more concrete evidence of benefits and limitations in regards to medication adherence.

### Do Smart Inhalers Increase Concordance With Prescribed Doses?

The previously mentioned studies used devices that collected data on the time of inhaler use, but this was not explicitly analysed for publication. It is therefore unclear if the inhaler doses were taken at the times intended by the prescriber. A further important issue with assessing concordance simply as the number of doses delivered by the inhaler is when an excess number of doses of medicine is dispensed in a single day. This may be either because an individual self-medicates with more preventative treatment as they feel unwell, or because they are “dose dumping” in an endeavour to appear compliant to healthcare professionals. In the relatively early trial from Charles et al. there were 53 instances amongst 110 participants of the use of at least 10 actuations of their ICS in 1 day, with a quarter of these occurring on the day of the clinic (19).

To understand the relationship between standard measures of adherence including dose use, and more recently available metrics such as doses taken within their prescribed time interval, Moran et al. (24) undertook a prospective observational study of 184 individuals with COPD. In this group, concordance with LABA-ICS as measured by dose use was 59%, but only 47% of doses were attempted within the scheduled dosing window. The same group also published a more extensive evaluation of an apparently overlapping study population (25) after hospital discharge. In this study, the average adherence (dose use) was higher but was again more than 10% greater than if the dose timing was taken into account.

The same INhaler Compliance Assessment (INCA) devices were used to establish if feedback could improve true concordance in severe asthma to a greater extent than intensive educational support (26). After 3 months of follow-up, those receiving smart inhaler feedback took more doses from their inhaler, but significantly fewer were in the prescribed dosing window (73 vs. 82%, *p* = 0.01). Once again, further large-scale studies are required to more accurately evaluate the relationship between smart inhalers and concordance with prescribed doses.

### Do Smart Devices Increase Inhaler Competence?

Advances in smart inhaler technology allow feedback to users on their inhaler technique with some devices. This allows the calculation of a true level of adherence (dose taken at the right time in the right manner). This value is well below those that might be recorded by medicine possession ratio or dose counting. For example, in the study of patients with post-discharge COPD, only 6% of individuals had >80% actual concordance with their LABA-ICS. Similar results have been found in mixed populations of airways disease (27).

In the study by Sulaiman et al. (26), smart inhaler feedback was found to be superior to intensive education in terms of the proportion of doses taken appropriately and on time (73 vs. 63%) due to lower rates of missed doses, technique errors, and overdoses. Hence, as demonstrated, smart devices have the potential to markedly improve inhaler competence. However, significantly more multicentre studies are required to demonstrate this on a larger scale and therefore influence clinical practice.

### Do Smart Devices Improve the Deployment of Action Plans?

Smart devices hold great promise as part of a system to automate asthma and COPD action plans, improving the speed and appropriateness of escalation of care for these conditions, and thus potentially reducing the occurrence of serious harm (28). Although promising multicentre research is being undertaken to produce this type of comprehensive solution (29), we are aware that no study was published which specifically addressed this review question. Future studies addressing this question will be vital in shedding light on the relationship between smart devices and effective action plan deployment.

## Discussion

Low concordance with inhaled medication use, timing, and technique has long been recognised to be one of the major obstacles in reducing harm from asthma and COPD in higher-income countries. Despite the tremendous potential impact of “smart-inhalers” on this aspect of airways disease management, a small volume of research studies has been undertaken in this area. In a large proportion of the studies that involve smart inhalers and particularly those with large sample sizes [e.g., the trials of as needed budesonide-formoterol (30)], the devices are deployed to measure the use and effect of a therapeutic intervention rather than being systematically studied in their own right.

Although the number of publications in this area is limited, in this review we highlighted that there appears to be robust evidence that the use of smart inhalers is associated with a substantial increase in the number of doses or preventer inhaler taken. This effect has been seen in paediatric and adult populations, in primary and secondary care, and in asthma and COPD. If an increase in concordance of the magnitude seen in these studies were replicated on a population level, it would be sufficient to have a major impact on serious adverse outcomes such as asthma deaths (31).

Smart inhalers also have clear potential in improving true concordance with prescribed therapy. However, data in this area are currently limited to one device type and thus further work is required to establish generalisability of these promising initial study findings. In the longer term, connected devices are likely to facilitate adherence to medical advice including action plans, though data are lacking in this area at present. These are central issues, but by no means the only research questions that remain outstanding in relation to smart inhalers. The acceptability of devices to users, the environmental impact of such a high volume production of disposable electronics, and the impact of the current lack of interoperability of systems also require systematic investigation.

Given the available randomised trial evidence, it is notable that smart inhaler use is not more clearly recommended in widely used guidance for asthma and COPD. For example, the latest GINA guidelines (5) make many “Grade D” recommendations based on the experience of the authors, but simply suggest smart inhaler use as an example of a potentially successful strategy to improve adherence.

The use of smart inhalers with dose reminders and feedback has a limited but supportive evidence base in relation to concordance with preventative therapy. A clearer focus on reducing harm will hopefully see further studies in this area and also being funded, and more thorough consideration in guidelines of the airways disease.

## Author Contributions

JB had the idea for the structure of the review. JB and CZ undertook literature searches, collated relevant publications, and contributed to writing the manuscript. All authors contributed to the article and approved the submitted version.

## Conflict of Interest

In the last 3 years, JB has undertaken educational presentations and advisory boards for companies that manufacture inhaled medicines, with fees going to charitable accounts (Astra Zeneca, Boehringer Ingelheim, Chiesi, GSK). He has benefitted from travel and accommodation relating to conference and educational meeting attendance (Boehringer Ingelheim, GSK). He has investigator initiated grants or involvement in research collaborations with “in kind” benefit (GSK, Novartis, Teva). The remaining author declares that the research was conducted in the absence of any commercial or financial relationships that could be construed as a potential conflict of interest.

## Publisher's Note

All claims expressed in this article are solely those of the authors and do not necessarily represent those of their affiliated organizations, or those of the publisher, the editors and the reviewers. Any product that may be evaluated in this article, or claim that may be made by its manufacturer, is not guaranteed or endorsed by the publisher.
